# High intrinsic hydrolytic activity of cyanobacterial RNA polymerase compensates for the absence of transcription proofreading factors

**DOI:** 10.1093/nar/gkz1130

**Published:** 2019-12-16

**Authors:** Amber Riaz-Bradley, Katherine James, Yulia Yuzenkova

**Affiliations:** 1 Centre for Bacterial Cell Biology, Biosciences Institute, Faculty of Medical Sciences, Newcastle University, Newcastle upon Tyne NE2 4AX, UK; 2 Life Sciences, Natural History Museum, Cromwell Road, London SW7 5BD, UK; 3 Department of Applied Sciences, Faculty of Health and Life Sciences, Northumbria University, Newcastle upon Tyne NE1 8ST, UK

## Abstract

The vast majority of organisms possess transcription elongation factors, the functionally similar bacterial Gre and eukaryotic/archaeal TFIIS/TFS. Their main cellular functions are to proofread errors of transcription and to restart elongation via stimulation of RNA hydrolysis by the active centre of RNA polymerase (RNAP). However, a number of taxons lack these factors, including one of the largest and most ubiquitous groups of bacteria, cyanobacteria. Using cyanobacterial RNAP as a model, we investigated alternative mechanisms for maintaining a high fidelity of transcription and for RNAP arrest prevention. We found that this RNAP has very high intrinsic proofreading activity, resulting in nearly as low a level of *in vivo* mistakes in RNA as *Escherichia coli*. Features of the cyanobacterial RNAP hydrolysis are reminiscent of the Gre-assisted reaction—the energetic barrier is similarly low, and the reaction involves water activation by a general base. This RNAP is resistant to ubiquitous and most regulatory pausing signals, decreasing the probability to go off-pathway and thus fall into arrest. We suggest that cyanobacterial RNAP has a specific Trigger Loop domain conformation, and isomerises easier into a hydrolytically proficient state, possibly aided by the RNA 3′-end. Cyanobacteria likely passed these features of transcription to their evolutionary descendants, chloroplasts.

## INTRODUCTION

Correct and fast copying of genomic sequence from DNA into RNA during transcription is vital for the faithful expression of genetic information. The elongation stage of transcription contributes significantly towards overall transcription efficiency. Arrested RNAP molecules are reactivated via hydrolysis of the 3′-end of the RNA. One of the major reasons for arrest is misincorporation, therefore hydrolysis serves as a proofreading reaction. Escape from transcriptional arrest via transcript hydrolysis is essential for efficient transcript elongation and cell viability (1). The mechanism of transcript hydrolysis is remarkably similar among all living organisms (2–4). In the absolute majority of organisms characterised so far, a weak intrinsic hydrolysis by RNAP is augmented by elongation factors, Gre in bacteria and TFIIS/TFS in eukaryotes/archaea (5–9). These proteins are not homologous between the two kingdoms of organisms but do share a general mechanism. This similarity suggests a functional convergence of proofreading factors and a strong incentive for an organism to encode them.

And yet, Gre/TFIIS factors are notably absent from one of the largest groups of bacteria, cyanobacteria (and several other smaller free-living taxons). These Gram-negative photosynthetic bacteria and evolutionary cousins of chloroplasts, are one of the oldest, most successful and widespread phylogenetic groups. Assuming cyanobacteria possess an evolutionary primitive mechanism of transcription, we can get a glimpse of an alternative way of supporting the fidelity and processivity of transcription, and perhaps the evolutionary reason for the acquisition of additional elongation factors in other lineages.

In all multisubunit RNAPs, transcript elongation and transcript hydrolysis are performed by the same, highly conserved active centre of RNAP. For all reactions, RNAP utilizes a two metal ion (Me^2+^) mechanism (10). The first Me^2+^ is held by an invariant triad of aspartate residues, and the second Me^2+^ is brought into the active centre by the substrate: nucleoside triphosphate (NTP), pyrophosphate or hydroxyl ion. Additionally, efficient catalysis of either phosphodiester bond formation or hydrolysis requires the correct folding of a flexible domain of the active centre, the Trigger Loop (TL), and its supporting domain, the Bridge Helix (BH) (6–9). The TL oscillates between closed (active) and open (inactive) conformations via intermediate conformations; most reactions do not require full TL opening (11). During RNA synthesis, closing of the TL stabilizes transition state of reaction, providing an induced fit mechanism of catalysis (12–14).

After RNAP incorporates an incorrect NMP by mistake, the 3′-end piece of RNA loses contact with the template, RNAP backtracks one base pair along the template, and the elongation complex becomes deactivated. Elongation resumes once the error-containing piece of RNA is cleaved out and a new, correctly paired RNA 3′-end is generated. The hydrolysis reaction following misincorporation is very efficient, due to the stabilization of elongation complexes in a 1 bp backtracked conformation (11). This general mode of transcriptional proofreading via transcript hydrolysis is similar among all characterized RNAPs (2–4). The TL participates in the hydrolysis reaction either by positioning of the reactants and stabilization of the transition state (12), or directly in some cases (15). The fascinating feature of intrinsic proofreading is the direct involvement of the 3′-end of a transcript in its own excision, resembling ribozymes (16–18). The involvement and the nature of a general base in catalysis is still a matter of continuing debate in the field (15,19).

It is generally accepted that intrinsic hydrolysis is not fast enough to correct transcriptional mistakes in real time in the cellular context, and that the modern proofreading mechanism relies on specialized protein factors. The best-studied member of the group is the prokaryotic protagonist, GreA of *Escherichia coli*. GreA is a member of a group of homologous proteins which bind at the secondary channel (hence their alternate name, secondary channel binding factors). This channel provides a route for substrate entry into the active centre. By inserting a coiled-coiled domain through the channel into the active centre, GreA flips the TL open and physically replaces it in the active site, thereby stopping elongation. Acidic amino acid residues at the tip of the coiled-coil domain of GreA stabilize the second Me^2+^ ion and coordinate a water molecule which greatly increases the efficiency of hydrolysis (17,20,21). As a result, GreA improves the fidelity of transcription by up to two orders of magnitude in some instances (22). Gre factors can also reactivate correctly paired complexes that have backtracked and arrested for various reasons (such as prolonged pauses) (23,24). Consequently, timely cleavage and reactivation of backtracked paused transcription complexes is a vital mechanism to remove stalled RNAPs out of the way of the replisome to avoid collisions, and to prevent the formation of traffic jams of RNAPs on actively transcribed genes in bacteria (25,26). Recently, the detailed structural pathway from backtracked elongation complex through Gre factor-dependent cleavage to the resulting reactivated elongation complex was visualized using Cryo-EM (27), supporting our understanding of the Gre-dependent hydrolysis mechanism. Secondary channel binding factors are not essential in laboratory conditions, but their loss is severely detrimental for the viability of all bacterial species tested, including *E. coli* and *Streptococcus pneumoniae*; their functional importance rises in different stress conditions (25,28).

Here, we studied the native RNAP of a widely used in research and biotechnology species of cyanobacterium, *Synechococcus* sp. PCC 6803 (*Ssp*RNAP). Most of our results were also reproduced with the RNAP of the distantly related *Synechococcus elongatus* 7942 (*Sel*RNAP), another model cyanobacterium, which allowed a generalisation of our findings for the whole group (See Supplementary Information). We found that cyanobacterial transcriptional fidelity is not severely compromised by the absence of proofreading factors, and that the level of *in vivo* mistakes in mature RNA is only fractionally higher than those of *E. coli*. *In vitro, Ssp*RNAP is not more accurate in substrate choice, yet proofreads transcription up to two orders of magnitude faster than *Ec*RNAP. We suggest that the *Ssp*RNAP active site tends to reside in a closed, hydrolytically-competent conformation. In cyanobacteria, the hydrolysis reaction is assisted by a general base, similarly to the Gre-stimulated reaction, and in contrast to *Ec*RNAP. An unexpected consequence of the cyanobacterial active site conformation is the suppression of transcriptional pausing.

## MATERIALS AND METHODS

### Strains and plasmids

*Synechocystis* sp. PCC 6803 strain was a gift from Prof Robinson, Durham University, UK; *Synechococcus elongatus* PCC 7942 was a gift from Prof Mullineaux, Queen Mary University of London; *E. coli* strain PGe74 (MG1655 *ΔgreAgreB*) was a gift from Dr Gamba, Newcastle University, UK. Plasmid pIA349 was a gift from Prof. Artsimovitch, Ohio State University, USA.

### Mutagenesis and purification of RNAPs

To generate mutations in *E. coli rpoC* gene, pRL663 plasmid encoding *E. coli rpoC* with C-terminal His_6_-tag under IPTG inducible promoter was used as the template for mutagenesis by QuickChange XL Mutagenesis Kit, ThermoFisher, according to the manufacturers’ protocol. WT and mutant constructs were transformed into the *E. coli* MG1655 *ΔgreAgreB* strain. Cell cultures were grown up to OD_600_ of 0.6, and 1mM IPTG was added to induce expression of plasmid-borne β’ for 3.5 hours. RNAPs were purified by Heparin (HiTrap Heparin column, GE Healthcare), Ni-NTA affinity (on HisTrap column, GE Healthcare) and ion exchange (ResourceQ column, GE Healthcare) chromatographic steps. *Synechocystis* sp. PCC 6803 was grown at constant light (100 μmol photons m^−2^ s^−1^) at 30°C in BG-11 medium. Harvested cells were disrupted using bead beater with 0.1 mm zirconia beads. After centrifugation at 15000 rpm for 20 min, followed by ultracentrifugation at 100 000 rpm for 1 h (to remove membrane fraction), lysate was loaded onto Heparin column (HiTrap Heparin column, GE Healthcare). RNAP was further purified using size exclusion (Superose 6, GE Healthcare) and ion-exchange chromatography (ResourceQ column, GE Healthcare) steps.

### Transcription assays

All transcription experiments were done at 30°C in transcription buffer containing 20 mM Tris–HCl pH 7, 40 mM KCl, 10 mM MgCl_2_, unless otherwise specified. Elongation complexes (ECs) were assembled and immobilized on streptavidin sepharose beads (GE Healthcare) as described (14,16). Sequences of the oligonucleotides used for the elongation complexes assembly are shown either in corresponding figures or Supplementary Figure S1. RNA was either kinased at the 5′-end using [γ-^32^P] ATP and T4 polynucleotide kinase (Fermentas) or labelled at the 3′-end after elongation complex assembly by incorporation of [α-^32^P] GTP (Hartmann Analytic), dictated by template, with subsequent removal of unincorporated nucleotide by washing beads with transcription buffer. To determine the rate of nucleotide addition, 1 μM NTP together with 10 mM MgCl_2_ (final concentrations), was added to the initial EC, reactions were incubated at room temperature and stopped by addition of formamide/8 M urea—containing loading buffer. Products were resolved by denaturing (8 M urea) 23% PAGE, revealed by PhosphorImaging (GE Healthcare) and analysed using ImageQuant software (GE Healthcare). The proportion of elongated RNA was plotted against time and fitted to a single exponential equation by using nonlinear regression in SigmaPlot software.

Misincorporation was initiated by simultaneous addition of 10 mM MgCl_2_, and 1 mM non-cognate NTP, reactions were kept at 30°C for the times indicated in the Figure 2A. To determine the rate of misincorporation, the proportion of complexes that undergone misincorporation (and subsequent cleavage) was plotted against time and fitted as above. Cleavage reactions were initiated by addition of 10 mM MgCl_2_ (final concentration), unless otherwise specified. Reactions were incubated at 30°C for the times indicated in the figures, and were stopped by addition of formamide/8 M urea—containing loading buffer. Products were resolved by denaturing (8 M urea) 23% PAGE, revealed by PhosphorImaging (GE Healthcare) and analysed using ImageQuant software (GE Healthcare). To determine the rate of phosphodiester bond hydrolysis, the proportion of the cleaved RNA was plotted against time and fitted to a single exponential equation using non-linear regression (14,16). To determine the *k*_M_ (Mg^2+^) for cleavage in mEC(A) and mEC(U), the reaction rates obtained at various MgCl_2_ concentrations were fit to the Michaelis–Menten equation (14,16). For activation energy calculations, cleavage rates in mEC(U) at 0, 5,10, 15, 20, 25, 30, 37, 42 and 45°C were calculated first and then plotted in ln *K* versus 1/*T* coordinates. Data were fit into the linear equation ln *K* = ln *A –* *E*_a_/*R*(1/*T*) using SigmaPlot software.

For elongation experiments on the IA349 template, an initial stalled complex EC37 was formed using 150 μM CAUC, 5 μM ATP, 5 μM CTP, 1.3 μM [α-^32^P] GTP (700 Ci/mmol) and biotinylated PCR-generated template DNA. After 2 min incubation, streptavidin sepharose (GE Healthcare) beads were added and incubated for additional 2 min, then washed twice with transcription buffer containing 200 mM NaCl and then twice with transcription buffer. Elongation was started by addition of 1 mM NTPs and 10 mM MgCl_2_.

### Phenotypic suppression of the temperature sensitive growth of the *E. coli ΔgreAgreB* strain

*Escherichia coli* strain MG1655 *ΔgreAgreB* (29) was transformed with pRL663 (30) based plasmids expressing either WT or mutant β’-subunits under an IPTG inducible bacterial promoter. Overnight cultures were diluted and grown until mid-exponential phase, then each culture was diluted up to OD_600_ 0.1. These initial cultures were further serially diluted, plated and incubated overnight either at 30°C or 37°C with addition of 0.1 mM IPTG to drive the expression of plasmid-encoded subunit.

### Next-generation sequencing and data analysis

Total RNA was isolated from mid-exponentially growing cultures of *E. coli* and *Synechocystis* sp. PCC 6803 as described in (31). Quality of RNA was checked by Agilent BioAnalyser, sample preparation and sequencing were performed by Vertis Ltd, essentially as described in (25), the only modification of the protocol is usage of PrimeScript, Clontech high fidelity reverse transcriptase. Dataset quality was assessed using FastQC (https://www.bioinformatics.babraham.ac.uk/projects/fastqc/) to ensure per base and per tile sequence quality. Raw reads were trimmed using fastx_trimmer (http://hannonlab.cshl.edu/fastx_toolkit/). Trimmed reads were aligned to genomes using Bowtie (32) allowing three mismatched bases and only unique alignments (-n 3 -m 1). *Escherichia coli* alignment used the NC_000913.3 reference genome and *Synechocystis* sp. *PCC* 6803 used the consensus derived from the in house sequencing data using CLC Workbench (https://www.qiagenbioinformatics.com/). Single base variations between the experimental *E. coli* strain and the reference genome were identified using samtools and bcftools (33). Error rate analysis was carried out in R using the BioConductor seqTools, seqInR and IRanges packages (34,35). Total error rates were calculated as the percentage of total reads with a mismatched base at each read position in the alignment, thresholded to a Phred quality score of <30. Specific error rates were calculated as the percentage of total reads with a specific mismatch, for example an A incorporated instead of a G (G > A misincorporation), at 7th position of each sequencing read, thresholded to a Phred quality score of <30. Ambiguous *N* bases and positions of single base variation were excluded from the error rate calculation. Raw and processed data were uploaded into GEO Database, under accession number GSE115135.

## RESULTS

### *Ssp*RNAP is not slow or accurate, but has high proofreading activity

**Figure 1.**
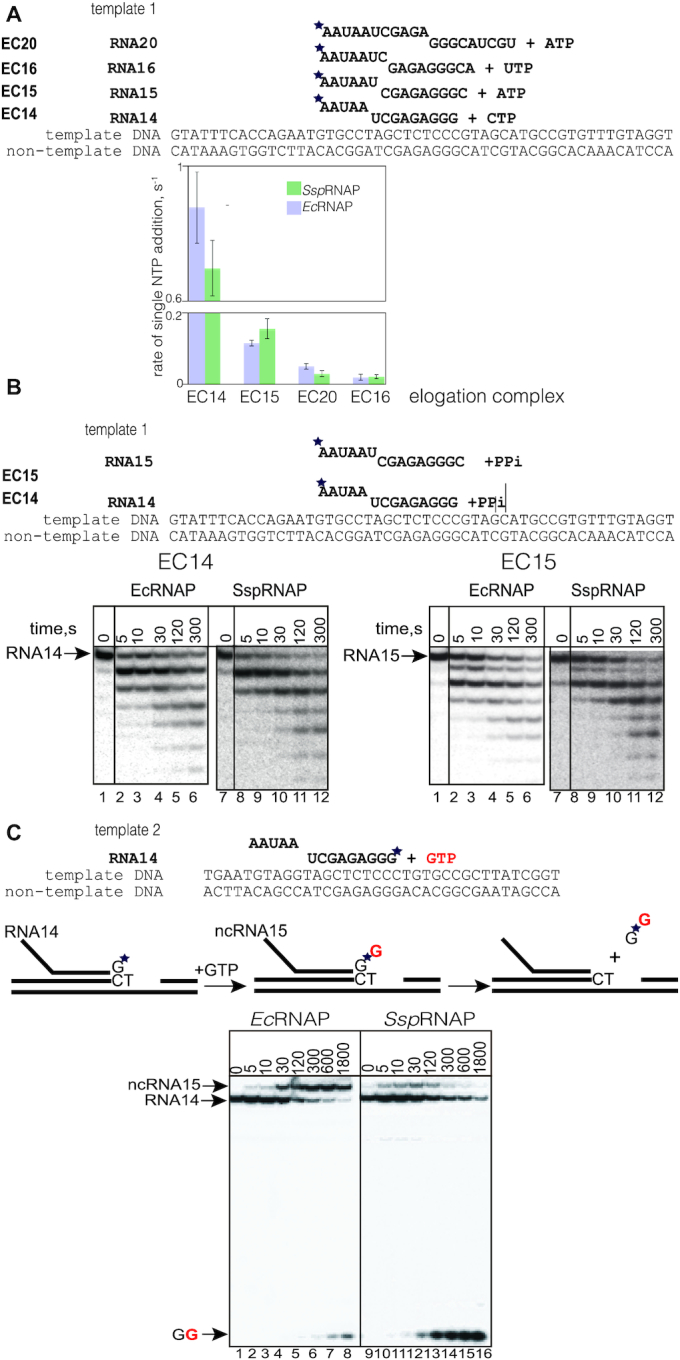
Rates of correct and incorrect substrate incorporation, and rates of pyrophosphorolysis are similar between *Ssp* and *Ec*RNAPs. (**A**) Rates of incorporation of correct NTPs are similar for *Ssp*RNAP and *Ec*RNAP. Rates of incorporation (s^−1^) of the single correct (1 μM) substrates by *Ssp*RNAP and *Ec*RNAP were compared in elongation complexes with 14 nt, 15 nt, 16 nt and 20 nt long RNA (EC14, EC15, EC16 and EC20) on template 1, are presented as bars on the graph. Scheme and sequence of RNA and DNA strands are above the graph. The elongation complexes were obtained by extension of initial RNA13, labelled with ^32^P at the 5′-end as indicated by the asterisk, upon NTPs addition. Error bars represent standard deviations from triplicate experiments. (**B**) Rates of pyrophosphorolysis are similar for *Ssp*RNAP and *Ec*RNAP. Kinetics of pyrophosphorolysis in EC14 and EC15 on template 1 using 250 μM pyrophosphate. (**C**) *Ssp*RNAP misincorporates substrates with same efficiency as *Ec*RNAP, and proofreads a mistake faster. Kinetics of misincorporation by *Ec*RNAP and *Ssp*RNAP, representative gel for misincorporation reaction of GTP instead of template-dictated ATP in EC14 on template 2. Schematics above the gel show elongation complex and reactions of misincorporation and subsequent hydrolysis with dinucleotide product release. Asterisk indicates that RNA is labelled at the 3′ end, which allows monitoring of misincorporation and proofreading simultaneously. Initial 14nt RNA (RNA14) after GTP misincorporation elongates to ncRNA15 (non-*c*orrect), then the 3′ incorrect dinucleotide piece of the transcript (GG) is cleaved out.

We assumed that cyanobacteria compensate for the proofreading factors’ absence by having either more accurate incorporation, or more efficient error proofreading (or possibly both). It has been suggested that cyanobacterial RNAP is a slow elongating enzyme (36), which could contribute to its accuracy by providing a longer time frame for correct substrate selection. We found that this is not the case, judging from single nucleotide addition experiments in *in vitro* assembled elongation complexes, ECs. As can be seen from plot in Figure 1A, the rates of substrate addition were comparable for *Ssp*RNAP and *Ec*RNAP in four elongation complexes on template 1.

The rates of the pyrophosphorolysis reaction, a direct reversal of nucleotide addition, were similar for the two enzymes in EC14 and EC15 (Figure 1B), meaning that catalytic rates do not differ between the *Ec*RNAP and *Ssp*RNAP. NTP addition proceeds exclusively from a post-translocated state, and pyrophosphorolysis from pre-translocated state. These experiments demonstrated that *Ssp*RNAP is at a similar equilibrium point between pre- and post-translocation states to that of *Ec*RNAP.

To test how readily *Ssp*RNAP incorporates incorrect substrates, we tested the kinetics of misincorporation of noncognate 1 mM NTPs, which is within the range of cellular concentration, into 14 nt long 3′-end labelled RNA in assembled elongation complex EC14 on template 2 (Figure 1C and Supplementary Figure S1). This set up allows simultaneous observation of both misincorporation and proofreading via dinucleotide cleavage. In the experiment shown in Figure 1C, RNAP was forced to incorporate GTP instead of template-dictated ATP. The rate of misincorporation, calculated as the rate of initial RNA14 transition into reaction products, was slightly higher for the *Ssp*RNAP (0.016 s^−1^ compared with 0.012 s^−1^ for *Ec*RNAP), suggesting that *Ssp*RNAP is not more accurate. Notably, however, the amount of erroneous transcript, *nc*RNA15 for *Ssp*RNAP was significantly lower at all time points, due to very efficient cleavage of the erroneous 3′-end dinucleotide, pGpG (compare the dinucleotide bands in lanes 6–8 with 14–16, Figure 1C). A similar effect on misincorporation and cleavage was observed for misincorporation of CMP instead of UMP (Supplementary Figure S2).

We conclude that *Ssp*RNAP has highly efficient proofreading activity, rather than highly accurate substrate incorporation. Therefore, it appears that efficient intrinsic hydrolysis is a primary compensatory mechanism for the Gre factors’ absence in cyanobacteria.

### Absence of Gre results in a moderate drop in the fidelity of transcription *in vivo*

**Figure 2.**
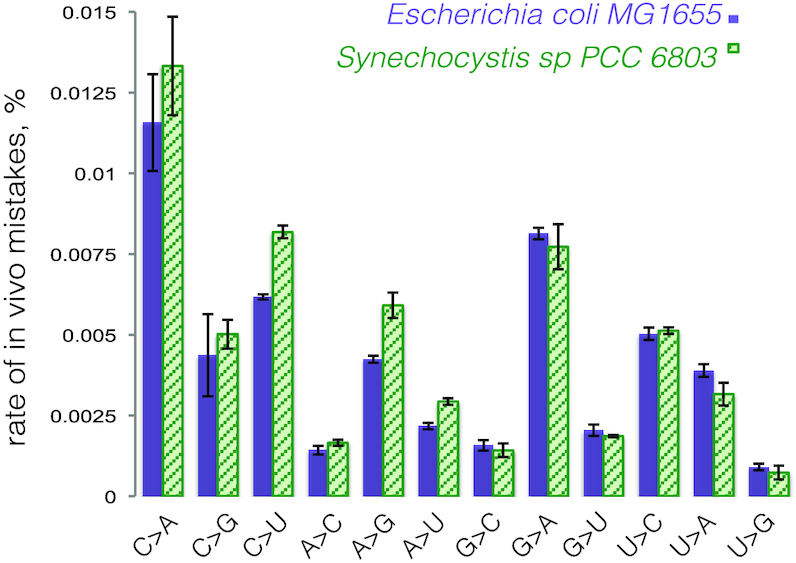
Rates of *in vivo* transcriptional mistakes are comparable between *E. coli* MG1655 and *Synechocystis* sp. PCC 6803. The graph shows percentages of total specific *in vivo* mistakes in mature transcripts for *E. coli* MG1655 and *Synechocystis* sp. PCC 6803, at specific positions of the sequencing read, calculated based on Next Generation Sequencing of total RNA from two species (See Methods for details). Error bars represent standard deviation from biological triplicates.

Is the hydrolytic activity of *Ssp*RNAP high enough to keep the rate of *in vivo* transcriptional mistakes at the level of *E. coli* where transcription fidelity is aided by Gre factors? To answer this question we assessed the levels of mismatches in the mature RNA *in vivo* by Next Generation Sequencing of the transcriptomes of *E. coli* and *Synechocystis* sp. 6803. To minimize the level of technical mistakes, we used the reverse transcriptase with highest fidelity available, which had been employed previously to determine the *in vivo* rates of transcriptional mistakes in *E. coli* (37). We compared the rate of mismatches at a particular position in the middle of the sequencing reads and found that levels of some base changes are indeed higher in *Synechocystis* (C to U, and A to G (Figure 2)), but at most increased to 125%. This increased level of *in vivo* mistakes in RNA, although not removing the probability entirely, argues against any additional unknown proofreading factors in *Synechocystis* sp. 6803.

### The molecular mechanism of fast *Ssp*RNAP hydrolysis

What is the molecular mechanism behind the fast hydrolysis exhibited by *Ssp*RNAP? Efficient hydrolysis requires a particular geometry of the reactants—the scissile phosphate bond, two Mg^2+^ ions, and attacking water. Since the 3′-end NMP of the RNA provides additional chemical groups to the active center, the characteristics of the reaction also depend on the nature of this NMP (16).

We investigated all elements of the hydrolysis mechanism using *m*isincorporated *e*longation *c*omplexes, mECs – elongation complexes where the 3′ end NMP of the RNA is non-complementary to the template base while the DNA template and non-template strands were fully complementary to each other. These complexes mimic the state of elongation complexes after misincorporation, which is one of the main targets of Gre factors in the cell. These elongation complexes are stabilised in a 1 bp backtracked conformation, which removes any input from backtracking into the rate of second phosphodiester bond hydrolysis (16).

**Figure 3.**
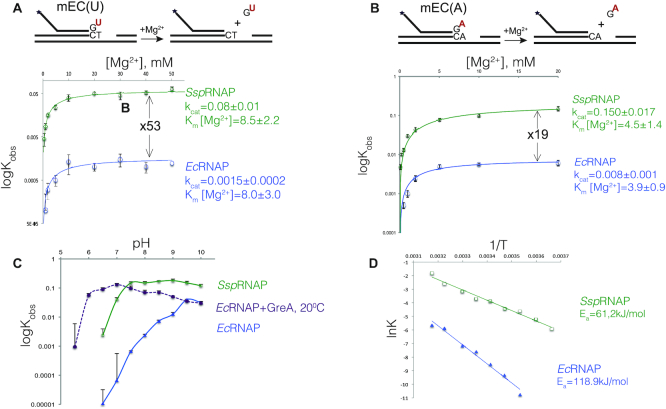
Molecular mechanism of fast transcript hydrolysis by *Ssp*RNAP. (**A** and **B**) Mg^2+^ dependencies of the hydrolysis rate of the penultimate phosphodiester bond in mEC(U) and mEC(A), respectively, by *Ec* and *Ssp*RNAPs. Schematics above the plots show the elongation complex structures and the hydrolysis reaction it undergoes; asterisk indicates that RNA is labelled at the 5′ end. Solid lines represent the graphical fits of data (using SigmaPlot software) to the Michaelis-Menten equation. The *k*_cat_ and *K*_M_ [Mg^2+^] (23) values are shown next to the plots. Error bars represent standard deviations from triplicate experiments. (**C**) pH profiles of second phosphodiester bond hydrolysis in mEC(A) complex for the intrinsic hydrolysis reaction by *Ec*RNAP, GreA assisted hydrolysis by *Ec*RNAP (at 20°C) and *Ssp*RNAP. The data points are averages of three independent experiments (standard deviation for each experimental point were within 10–15% value). (**D**) Arrhenius plots for hydrolysis reaction in mEC(U) complex by *Ec*RNAP and *Ssp*RNAP, graphical fits of lnK to 1/T data to linear equation are shown as a straight line, apparent activation energy calculated from equation ln *K* = ln *A* – *E*_a_/*R*(1/*T*) is shown on the plot. The data points are averages of two independent experiments.

We tested elongation complexes of 15 nt RNA with either U at the 3′- end (mispaired with template T) or A (mispaired with template A), mEC(U) and mEC(A) respectively (schemes of the reaction are above the graphs on Figure 3A, B and sequences on Supplementary Figure S1). In these elongation complexes, the *K*_M_ for Mg^2+^ was similar for *Ssp*RNAP and *Ec*RNAPs at pH 7.9 in both cases, but *k*_cat_ values for *Ssp*RNAP were 30 and 53 times higher than *Ec*RNAP for mEC(U) and mEC(A), respectively. These results suggest that the increased rate of hydrolysis does not come from the stabilization of the second Mg^2+^ ion in the active site (the mechanism proposed for Gre factors), which is consistent with both enzymes having conserved amino acid residues in the vicinity of the catalytic Mg^2+^ ions.

Hydrolysis requires deprotonation of water, and its efficiency depends on the pH of the reaction. The pH-dependence profile of *Ec*RNAP’s rate of intrinsic hydrolysis was different from both *Ssp*RNAP intrinsic hydrolysis and from GreA-assisted hydrolysis by *Ec*RNAP on mEC(A) (Figure 3C). For *Ec*RNAP in the range of pH 6.5 to 9.7, the dependence is log-linear with a gradient of ∼0.9, most likely reflecting water ionization. In contrast, both *Ssp*RNAP and GreA-dependent *Ec*RNAP reactions behave somewhat similarly—the graphs quickly plateau, although at different pH values. We suggest that *Ec*RNAP does not provide a general base for water activation, in agreement with the work of Mishanina *et al.* (19). In contrast, in both *Ssp*RNAP and GreA-dependent reaction, a general base which activates water is apparently involved. For the GreA-dependent hydrolysis, the p*K*_a_ is below 5.5, for *Ssp*RNAP, the p*K*_a_ is ∼6.8. The initial slope of the curve is >1, suggesting more than one group involvement. These results are in line with previous work on *T. aquaticus* RNAP, where a general base was provided in some instances in the form of the Trigger Loop His1242 residue (15).

By analysing temperature dependence of the hydrolysis reaction in mEC(U), we found that the activation energy of the reaction for *Ssp*RNAP is ∼2× lower compared to *Ec*RNAP (Figure 3D), suggesting easier isomerization into a reactive conformation. The same results were observed for mEC(A) (Supplementary Figure S5).

### Long range backtracking is not enhanced in cyanobacteria

**Figure 4.**
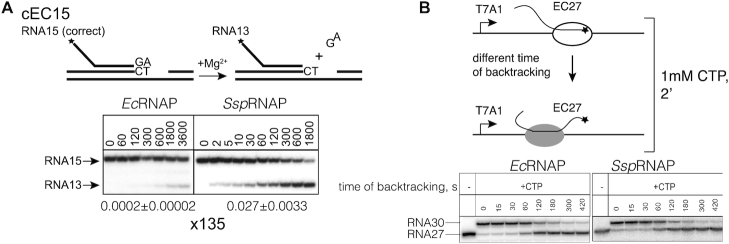
*Ssp*RNAP backtracks faster than *Ec*RNAP for 1 bp, but not for long distances. (**A**) Kinetics of hydrolysis of RNA15 in the correct elongation complex (scheme above the gel) on template 2 by *Ssp*RNAP and *Ec*RNAPs with rates of reaction underneath the gel calculated after fitting the data into exponential equation by SigmaPlot software. (**B**) Backtracking on T7A1 promoter template by *Ssp*RNAP and *Ec*RNAP. Elongation complex EC27 prone to backtracking was formed and incubated at 37°C at pH 6.0 for the indicated periods of time to allow backtracking to happen. Remaining activity was assessed by the ability of complexes able to elongate RNA to position 30 after addition of 1 mM CTP.

Misincorporated complexes are not the only targets of Gre factors. The other targets are correctly paired elongation complexes that are left in a backtracked state after undergoing arrest or a pause for various reasons (23,38). For these complexes, the speed of entering the backtracked state contributes to the overall rate of reaction. Does *Ssp*RNAP backtrack faster by 1 bp in a correctly paired elongation complex? We analysed the hydrolysis of a transcript in a correctly paired elongation complex with 15 nt long RNA with A at the 3′-end, cEC15 (Figure 4A). Indeed, the overall reaction in the correctly paired transcript in EC15 was faster in *Ssp*RNAP, and the difference in comparison to *Ec*RNAP was larger than in misincorporated complexes (Figure 4A)—135 fold higher in correctly paired elongation complex compared to 30 times in misincorporated mEC(A) (Figure 3B). Similarly fast RNA hydrolysis in the correct elongation complex was observed for RNAP of another cyanobacterium, *Synechococcus elongatus* 7942 (Supplementary Figure S3). We suggest that this higher difference is due to input from faster isomerization into a 1 bp backtracked state by cyanobacterial RNAP, probably assisted by 3′-end of the RNA, but this then raises the question of whether cyanobacterial RNAP backtracks faster in general.

To compare the ability of the *Ssp*RNAP and *Ec*RNAP to backtrack over longer distances we used a well-characterised, prone to backtracking elongation complex with 27 nt long RNA, formed on a linear DNA template containing the T7A1 promoter. Prolonged incubation of this elongation complex at 37°C typically leads to accumulation of backtracked inactive complexes (39) (Figure 4B). We monitored transition into a backtracked state by observing a progressive loss of ability of RNAP to extend 27 nt RNA upon addition of NTP substrates. *Ec*RNAP and *Ssp*RNAP were allowed to backtrack for the time intervals indicated in Figure 4B and then supplied with 1 mM CTP to elongate RNA in complexes still active from 27 nt to 30 nt long. The fraction of active complexes decreased similarly over time for both enzymes hence we concluded that beyond 1 bp *Ssp*RNAP moves backwards at the same rate as *Ec*RNAP (Figure 4B). In other words, only 1 bp backtracking, associated with proofreading, is specifically increased in cyanobacteria.

Which parts of the RNAP active site are responsible for efficient cleavage? The TL and supporting it BH have both been previously shown to influence transcript hydrolysis (15,19,40). There is also input from the 3′-end of the RNA (41). All of these are flexible players. Therefore, it is very probable that the conformation and dynamics of these domains are responsible for the efficient transcript hydrolysis characteristic for *Ssp*RNAP. We hypothesized that this efficiency has been achieved by the strategic placement of cyanobacteria-specific amino acid residues in the ‘hinges’ of the TL and/or BH.

### Cyano-specific amino acid residues in the Trigger Loop and Bridge Helix stimulate hydrolysis by *Ec*RNAP and suppress the phenotype of the Δ*greA*Δ*greB* strain

**Figure 5.**
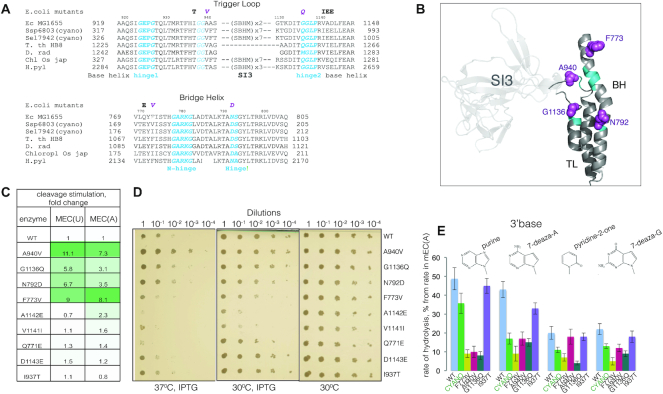
Cyano-specific amino acid residues in the TL and BH contribute to the fast transcript hydrolysis by *Ssp*RNAP. (**A**) Alignment of the amino acid sequences of the Trigger Loop and part of the Bridge Helix of *Synechocystis sp* PCC 6803, *Escherichia coli* MG1655, *Thermus thermophilus* HB8, *Oryza sativa* chloroplast and *Bacillus subtilis*. (**B**) Structure of the *E. coli* RNAP TL and BH domains from PDB 5IPM. Amino acid residues whose substitutions stimulated transcript hydrolysis are shown in magenta. Flexible hinges and additional GG motif in TL are shown in cyan. (**C**) Stimulation of transcript hydrolysis by the changes to cyano-specific amino acid residues in *Ec*RNAP in mEC(U) and mEC(A). Table lists the fold changes of the rates of hydrolysis by the mutant RNAPs in comparison to the WT *Ec*RNAP. (**D**) Expression of mutant *rpoC* genes coding for substitutions A940V, G1136Q, and N792D supress the growth defect of the *E. coli* MG1655 *ΔgreAgreB* strain. Serial dilutions were plated on Petri dishes and grown overnight at 30°C, 30°C with IPTG, or at 37°C with IPTG. (**E**) Effect of changing the chemical groups of the 3′-end RNA base (base structures on top of plot) on hydrolysis rate by WT *Ec*RNAP (sky blue), *Ssp*RNAP (grass green) and mutant *Ec*RNAPs with substitutions F773V (mustard), A940V (hot pink) G1136Q (malachite) and I937T (violet). The plot represents residual activity percentage in elongation complexes with 3′-end RNA modified bases in comparison to canonical base in mEC(A). The error bars represent standard deviation from triplicate data points.

The trigger loop consists of two helical parts separated by N-terminal and C-terminal ‘hinges’ and the SI3 insertion, present in cyanobacteria and *E. coli* (Figure 5A and B). The amino acid sequence corresponding to N-terminal part of the TL is the same for both *Ssp*RNAP and *Ec*RNAP, however several cyanobacteria-specific amino acids can be found in the unstructured region and in the C-terminal base helix of the TL (Figure 5A and B). To investigate if any of these amino acid residues contribute to efficient hydrolysis, we tested mutant *Ec*RNAPs whose native amino acid residues were changed to the cyano-specific ones in the TL (*E. coli* numbering)—I937T, A940V, A941F, K1132G, T1135V, G1136Q, V1141I, A1142E, D1143E and F1145L (Figure 5A). Rates of RNA hydrolysis were analysed in the assembled misincorporated elongation complexes mEC(A) and mEC(U) (Supplementary Figure S1). Most substitutions did not affect the rate of hydrolysis (Figure 5C and Supplementary Table S1, which also includes additional data on mEC(C)). However, two changes, A940V and G1136Q, increased the rate of hydrolysis by mutant *Ec*RNAP by 7–11-fold and 3–6-fold respectively, in both mECs. The amino acid residues at these positions are too far from 3′-end of RNA to participate in cleavage reaction directly. Notably, A940V is located next to double glycines in the N-terminal part of TL, and the G1136Q substitution is in the C-terminal glycine hinge of the TL. Both of these substitutions have the potential to affect folding dynamics of the TL.

Since folding of the TL proceeds in concert with the BH, and changes in the BH affect transcript hydrolysis (40), we looked for additional cyanobacterial-specific amino acid substitutions in the BH. The most conspicuous change is F773V, located close to N-terminal glycine hinge of the BH (Figure 5A). This substitution is severely detrimental to the growth of *E. coli* (42). F773V *Ec*RNAP increased the rate of the hydrolysis reaction on both mEC(A) and mEC(U) by 8–9-fold (Figure 5C). Another BH substitution, N792D, increased the hydrolysis rate 3.5-6.5-fold, while Q771E did not have an effect on hydrolysis rate (Figure 5C).

Could mutant RNAPs with increased proofreading efficiency suppress the temperature sensitive phenotype of an *E. coli* strain with both GreA and GreB factors deleted (24)? To address this question we expressed mutant (F773V, N792D, I937T, A940V, D1143E, Q771E, G1136Q, V1141I, A1142E) or WT β’-subunits from a pRL663 plasmid in the Δ*greA*Δ*greB* MG1655 strain of *E. coli*, plated and grew culture dilutions on solid media at either the permissive (30°C) or the nonpermissive (37°C) temperature with addition of IPTG to induce expression of the mutant subunits. Mutants with increased transcript hydrolysis efficiency (in particular A940V) were able to moderately promote growth in comparison to the WT and to other neutral mutants (Figure 5D). The lack of suppression by some mutants could be due to their own growth phenotypes, such as for F773V, which has been previously characterized as generally detrimental for viability of *E. coli* (43). Similarly, mutants A1142E and V1141I affected cell viability in general.

The 3′-end of the RNA contributes to the efficiency of its own hydrolysis reaction by providing additional coordination groups to water and Mg^2+^ ions (16). Change or removal of these 3′-base reduces the efficiency of hydrolysis. We found that *Ssp*RNAP and hydrolytically proficient, but not hydrolytically neutral, *Ec*RNAPs mutants are more sensitive to chemical modifications of the 3′-base. As can be seen from Figure 5E, changing 3′-adenine in a mEC(A) to a purine, pyridine-2-one, 7-deaza-A, or 7-deaza-G has a greater effect on the hydrolysis rate (Figure 5E) of *Ssp*RNAP, and of F773V, A940V and G1136Q *Ec*RNAPs. We suggest that a greater reduction of rate is related to a stronger original mechanism and that a specific folded TL conformation provides some interaction with mismatched 3′-end of RNA, as was proposed by Larson *et al.* (44).

### *Ssp*RNAP inefficiently recognises pausing signals

**Figure 6.**
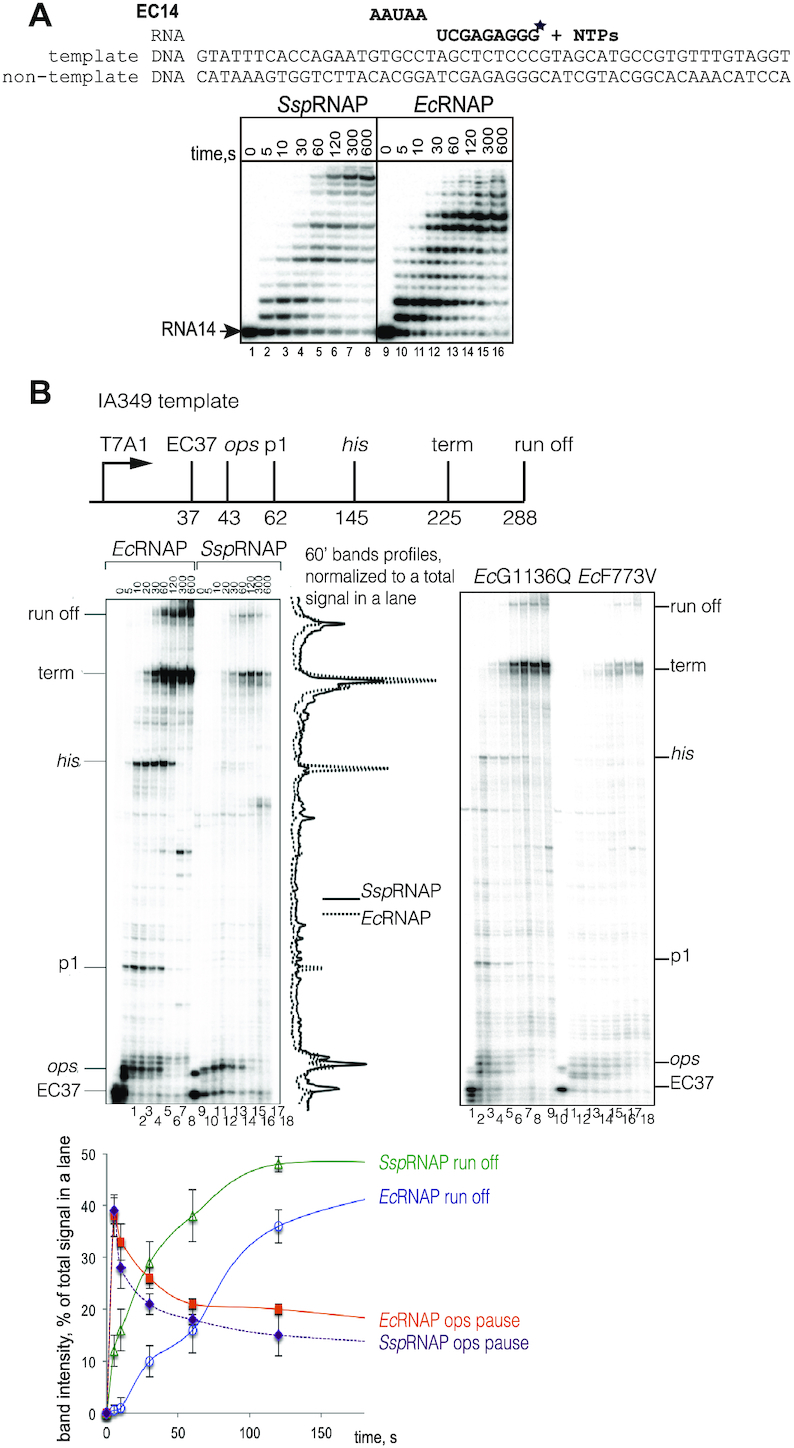
*Ssp*RNAP is less prone to pausing than *Ec*RNAP. (**A**) Kinetics of elongation for EC14 assembled on template 1 using all four NTPs for *Ssp*RNAP and *Ec*RNAP. Scheme and sequence above the gel, asterisk indicates the position of ^32^P label. (**B**) Single round elongation on IA349 template which encodes the T7A1 promoter, known pause sites, and terminator, shown on the scheme of the template above the gel images. Left gel – initial stalled elongation complex EC37 was formed with *Ec*RNAP and *Ssp*RNAP, then chased with all four NTPs. To the right of the gel image, superimposed traces of the 60s bands for the RNAPs were generated by ImageQuant software and normalized to the total amount of radioactivity in the lane. Graph below the gel images shows kinetics of run-off product accumulation and fraction of complexes paused at ops pause sequence for *Ec*RNAP and *Ssp*RNAP as a percentages from total radioactivity in the lane. Right gel shows kinetics of single round elongation on IA349 template performed similarly with mutant *Ec*RNAPs with substitutions G1136Q and F773V.

Pausing of RNAP during elongation is accompanied by TL opening (12,13,45,46). We hypothesized that pausing efficiency might be lower for *Ssp*RNAP due to the tendency of its active site to reside in a closed conformation. In the experiment in Figure 6A, we performed kinetics of transcript elongation in an assembled elongation complex with 14 nt long RNA, EC14, upon addition of a low concentration of all four NTPs. Indeed, the propensity of *Ssp*RNAP to make fewer ubiquitous pauses during elongation, and to reach the end of template faster, is evident from Figure 6A.

To test if the recognition of regulatory pausing and termination signals was similarly affected, we used the well-characterised template IA349, which encodes an *ops*, *his*, an additional pause p1, and a rho-independent terminator sequence downstream of a T7A1 promoter (scheme in Figure 6B). On a linear PCR-generated DNA template, after making a stalled elongation complexes with RNA37 in a subset of NTP substrates (Figure 6B, left gel image, lanes 1 and 10), the kinetics of elongation at low concentration of NTPs were analysed. *Ssp*RNAP has an altered pausing pattern in comparison to *Ec*RNAP (Figure 6B, compare the normalised traces from the 60-s time points). *Ssp*RNAP fails to recognise *his* or p1 pauses; the efficiency of termination is also decreased. Similar behaviour is displayed by β’F773V and β’G1136Q *E. coli* mutant RNAPs (Figure 6B, right gel image), implying that amino acids in these positions possibly determine the *Ssp*RNAP’s pausing phenotype in general. Consistent with our findings, β’F773V was previously characterised as pause-resistant, and a different mutant in the 1136 position, β’G1136S, as fast elongating (42,43,47). The only pause efficiently recognised by *Ssp*RNAP is the *ops* pause (plot with kinetics of *ops* pausing below gels), previously characterized as initially pre-translocated (38,48), and then backtracked (49). This result asserts once more that *Ssp*RNAP’s equilibrium between pre- and post-translocation states is similar to that of *Ec*RNAP.

Resistance to pausing, which we assume contributed to lower termination efficiency, might additionally suggest that *Ssp*RNAP is more resilient to the recently proposed RNAP conformational change, ‘swivelling’, which allosterically prevents TL folding (50) and induces pausing. Altogether, our results suggest that *Ssp*RNAP is less responsive to diverse pausing signals, and that transcription elongation in cyanobacteria might be a more continuous process in general (see also Discussion) in comparison to that of *E. coli*. This property might further alleviate the need for proofreading factors in cyanobacteria.

## DISCUSSION

Here, we found that cyanobacterial RNAP possesses a very efficient intrinsic proofreading mechanism. This proofreading mechanism is potent enough to keep the rate of *in vivo* transcriptional mistakes in *Synechocystis* sp. PCC 6803 at a level only fractionally higher than that of *E. coli*, which is still easily tolerated. Apparently, cyanobacterial RNAP efficiently performs the functions which are delegated to the Gre/TFIIS factors in other taxons. In this work we identified the aspects of this mechanism.

Gre factors increase the affinity for the catalytic metal ion (20) and activate the attacking water molecule (17) in the hydrolytic proofreading reaction. Affinity of the cyanobacterial enzyme for magnesium is not increased compared to *E. coli* RNAP, consistent with conservation of amino acid residues in the close vicinity of catalytic magnesium. However, water deprotonation by *Ssp*RNAP is assisted by a general base in a similar fashion to GreA (17) (general base was provided in some cases in *T. aquaticus* RNAP reaction (15)). For the GreA-dependent hydrolysis, the p*K*_a_ is below 5.5, which most likely corresponds to the p*K*_a_ of the active site glutamate (p*K*_a_ 4.1). For *Ssp*RNAP, the p*K*_a_ is ∼6.8, suggesting either a histidine residue (p*K*_a_ 6.0) or a phosphate group of the transcript (p*K*_a2_ 7.2) involvement. There is a possibility of more than one group participation, since the slope of the corresponding curve on Figure 3C is >1. In contrast, in *E. coli*, we did not detect the participation of a general base in hydrolysis, in agreement with the earlier suggestion of Mishanina *et al.* (19).

We propose that the cyanobacterial RNAP active site isomerises into a 1 bp backtracked hydrolytically proficient conformation with ease, reflected in a lower activation energy of the hydrolytic reaction compared to *Ec*RNAP. Notably, the activation energy barrier of *Ssp*RNAP cleavage is lowered down to almost exactly of that of Gre-stimulated *E. coli* cleavage determined by Miropolskaya *et al.* (51). Importantly, although isomerization of *Ssp*RNAP into a 1 bp backtracked state is very efficient, longer backtracking has a similar rate to *Ec*RNAP, hence there is no general propensity of cyanobacterial RNAP to move backwards and, therefore, no risk of frequent elongation interruptions. Perhaps, there are two separate thermodynamic or physical routes for 1 bp backtracking vs longer backtracking, and only the former is different in cyanobacteria.

Another aspect of the cyanobacterial hydrolysis mechanism is a different conformation or flexibility of the Trigger Loop and Bridge Helix domains, we assume. Four amino acid residues of these domains, specific to *Synechocystis* sp. 6803, when introduced into *Ec*RNAP significantly (3-11 times) increased the rate of hydrolysis, with magnitude decreasing in the order A940V > F773V > G1136Q > N792D. Cyanobacteria and chloroplasts have non-*E. coli*-specific amino acid residues at corresponding positions (Figure 5A). The A940V change, never reported before, resulted in the highest acceleration of *in vitro* hydrolysis and the best suppression of a temperature sensitive phenotype of the double *greAgreB* deletion *E. coli* strain. The F773V substitution, toxic for *E. coli*, was never reported to affect hydrolysis. At position 1136 different changes (to S and M) were shown to increase rate of hydrolysis by *E. coli* and *D. radiodurans* RNAPs (28,47), consistent with our results. Although these residues are located too far from the active site to directly participate in the reaction, they might change the conformation and the flexibility of the TL and BH. G1136 is located in the hinge region of the TL and A940V is next to a double glycine motif at the tip of the TL (Figure 5B, flexible parts are highlighted in cyan). We suggest that these residues stabilise a specific closed state of the TL, perhaps accompanied by a particular RNA 3′-end conformation. In support of our hypothesis, substitutions F773V and G1136S in *E. coli* were modelled to stabilize a closed TL (52,53), and recently were shown to stabilize a 1 bp backtracked state (54). Similarly, residue N792 is located in the flexible hinge region of the BH, and mutants at this position were predicted to fix the BH in a particular conformation (55,56). We hypothesize that the same residues which stabilize the hydrolytically proficient state in the absence of substrate also prevent ubiquitous pausing during active elongation, perhaps via resistance to conformation changes, e.g. ‘swivelling’.

Regulatory RNAP pausing, on the other hand, is a major regulatory mechanism in bacteria and eukaryotes which couples transcription with other cellular processes (57). It is unlikely that cyanobacteria lack this regulatory possibility entirely, they may react to different signals or employ as yet unknown regulatory factors. Notably, hairpin dependent pauses are not recognised, which might have implications for intrinsic termination. Perhaps, pauses like *ops*, which are very efficiently recognised by *Ssp*RNAP, play regulatory roles *in vivo* (a simple search finds approximately 100 *ops*-like sequences in the genome of *Synechocystis* sp. PCC 6803).

We cannot completely rule out input from other domains of RNAP or 3′-end of the RNA into efficient proofreading and pause resistance; additional specific features may contribute to cyanobacterial RNAP protein dynamics, such as the physical location of the catalytic aspartate triad and TL/BH modules on two separate proteins due to the split of β’subunit, and the presence of a much larger SI3 insertion.

The high efficiency of hydrolysis and fast elongation are common for two distantly related species of cyanobacteria, *Synechocystis* sp. PCC 6803 and *Synechococcus elongatus* 7942, suggesting that these are general features of cyanobacterial transcription. Plant chloroplasts (descendants of ancient cyanobacteria), and other groups that lack proofreading factors, might share these features.

What are the physiological benefits of cyanobacterial reliance on intrinsic proofreading? It solves the problem of time-requirement for the recruitment of proofreading factors, and the cyanobacterial set up is reminiscent of eukaryotic RNAPs I and III, which carry out proofreading activity on one of the subunits (58). Notably, the hydrolytic activity of *Ssp*RNAP decreased with decreasing temperature at a slower rate than in *E. coli*, and was still apparent even at zero degrees, suggesting that in low winter temperatures intrinsic proofreading is still active in cyanobacteria when protein diffusion is slow (Supplementary Figure S4).

We assume that Gre factors were never acquired by cyanobacteria in evolution. Transcript cleavage factors are not conserved between the Archaea-Eukarya and Bacteria (59). According to the recently published tree of life, cyanobacteria belong to a deep phylum stemming directly from a common ancestor of Archaea-Eukarya and Bacteria, meaning that they might have branched out before proofreading factors acquisition (60).

Alternatively, Gre factors might have been lost after they became dispensable. The ultimate cellular role of Gre factors is to alleviate conflicts between the replication and transcription machineries (26), which may occur less frequently in cyanobacteria due to circadian regulation and polyploidy. In photosynthetic cyanobacteria, replication is thought not to coincide in time with the main peak of transcription (61). RNAP and the replisome might not operate on the same copy of genome in polyploidal cyanobacteria (e.g. *Synechocystis* sp. 6803 has >10 copies of its genome in a cell (62)). Hypothetically, Gre factors could have become detrimental because they can potentially bring a wrong metal ion, such as iron (very abundant in cyanobacteria (63)), into the RNAP active site which, when combined with reactive oxygen species generated by the electron transport chain, leads to protein damage (64). Gre factors interference with both nucleotide excision (65) and double strand break repair (66), are another possible incentives for their loss from cyanobacteria, which have a high level of genomic DNA photo-damage. Acquisition of the extremely large SI3 (∼650 aa) could have also led to occlusion of the Gre binding site and sped up the loss.

## DATA AVAILABILITY

Data generated from analysis of NGS in both raw and processed form are available at GEO Database, under accession number GSE115135.

## Supplementary Material

gkz1130_Supplemental_File
