# Supplementary material for: High intrinsic hydrolytic activity of cyanobacterial RNA polymerase compensates for the absence of transcription proofreading factors
Source: Nucleic Acids Res. 2019 Dec 16;48(3):1341–52. doi: 10.1093/nar/gkz1130 (PMC7026648; doi:10.1093/nar/gkz1130)
Supplement: gkz1130_Supplemental_File [file gkz1130_supplemental_file.pdf]

template 2

cEC(A)

```
                         AAUAA
                              UCGAGAGGGA*
RNA15(A)
template DNA         TGAATGTAGGTAGCTCTCCCTGTGCCGCTTATCGGT
non-template DNA     ACTTACAGCCATCGAGAGGGACACGGCGAATAGCCA
```

mEC(A)

```
                        *AAUAA
                              UCGAGAGGG A
RNA15(A)
template DNA         TGAATGTAGGTAGCTCTCCCCGTGCCGCTTATCGGT
non-template DNA     ACTTACAGCCATCGAGAGGGGCACGGCGAATAGCCA
```

mEC(U)

```
                        *AAUAA
                              UCGAGAGGG U
RNA15(U)
template DNA         TGAATGTAGGTAGCTCTCCCGGTGCCGCTTATCGGT
non-template DNA     ACTTACAGCCATCGAGAGGGCCACGGCGAATAGCCA
```

mEC(C)

```
                        *AAUAA
                              UCGAGAGGG C
RNA15(C)
template DNA         TGAATGTAGGTAGCTCTCCCAGTGCCGCTTATCGGT
non-template DNA     ACTTACAGCCATCGAGAGGGTCACGGCGAATAGCCA
```

Fig. S.1. Structure of elongation complexes based on template 2, and sequences of nucleic acid oligonucleotides used for their assembly. Asterisk indicates that RNA is labelled at the 5' - end by kinasing oligonucleotide using polynucleotide kinase A and [γ- P^{32}] ATP.

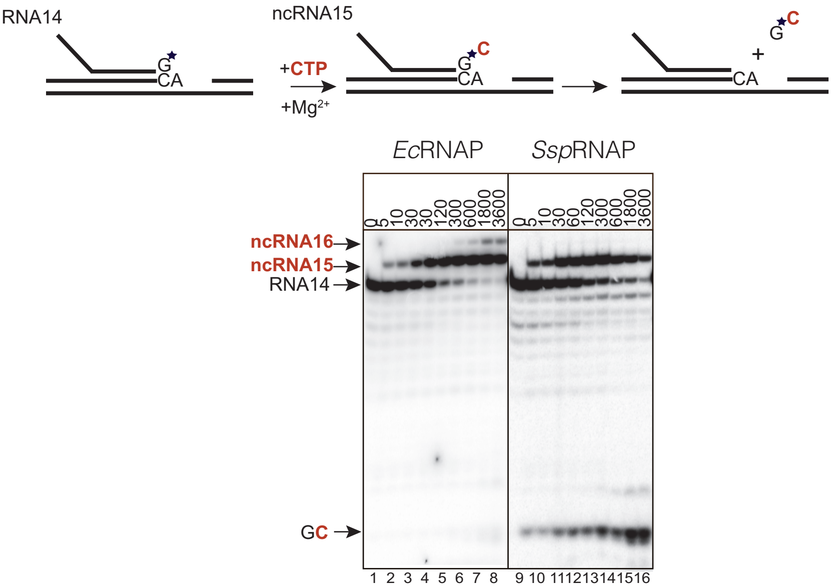

Fig. S. 2. Kinetics of misincorporation of CTP instead of UTP dictated by template with concomitant proofreading reaction - cleavage of the GC 3' dinucleotide portion of the transcript by EcRNAp and SspRNAP. Above the gel is the scheme of reaction; asterisk indicates that RNA is labelled at the 3' end by incorporation of radiolabelled GTP into the transcript. *Ssp*RNAP).

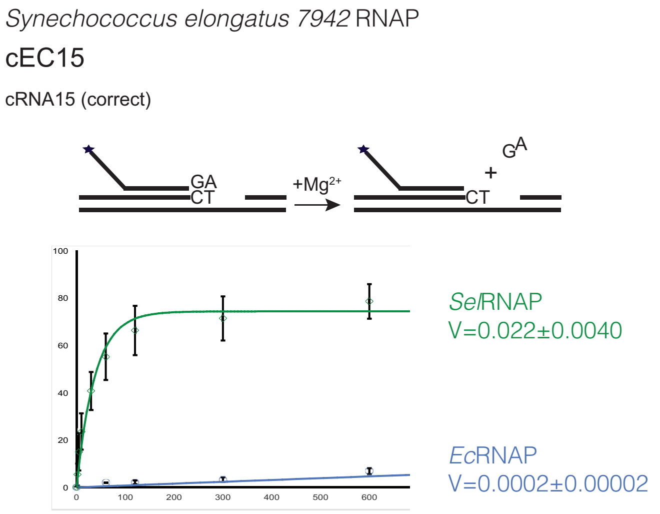

Fig. S.3. Kinetics of transcript hydrolysis in the correct elongation complex cEC(A) (Fig. S1) by purified RNAP of different cyanobacterium, Synechococcus elongatus, *Sel*RNAP. Above the gel is the scheme of reaction; asterisk indicates that RNA is labelled at the 5' - end by kinasing 13 nt RNA oligo using polynucleotide kinase A and [γ- P^{32}] ATP. After assembly into elongation complex, initial RNA13 was extended to RNA15 with addition of ATP and GTP. The graph fits of data to the exponential equation are shown as solid lines, *Ec*RNAP (blue) and *Sel*RNAP (green).

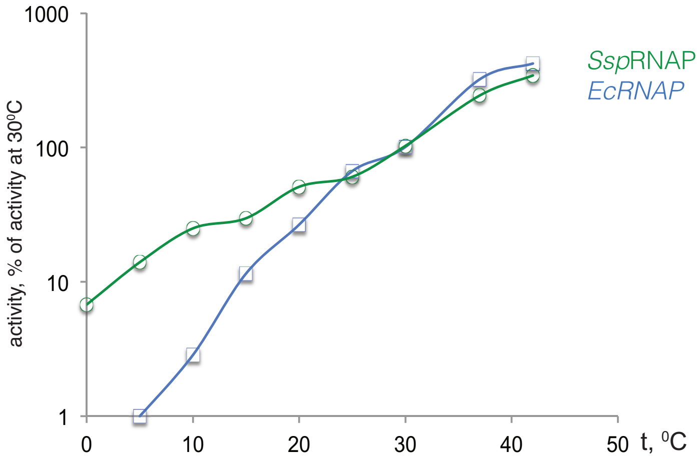

Fig. S.4. Temperature –dependence of rate of hydrolysis reaction in mEC(U) by *Ec*RNAP and *Ssp*RNAP.

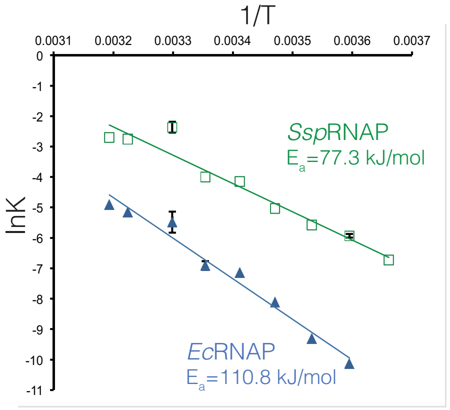

Fig. S.5. Activation energy for RNA hydrolysis in mEC(A) elongation complex by *Ec*RNAP and *Ssp*RNAP. . Arrhenius plots for *Ec*RNAP and *Ssp*RNAP, graph fit of lnK to 1/T data to linear equation are shown as straight line, apparent activation energy calculated from equation $lnK=lnA-E_a/R(1/T)$ is shown on the plot. The data points are averages of two independent experiments.

Table S1. Rates of transcript hydrolysis in mEC(A) and mEC(U) by WT and mutant *Ec*RNAPs with indicated amino acid changes in β' subunit at 30^0C and 10 $MgCl_2$.

RNAP	mEC(U) hydrolysis rate, s-1	Standart deviation	mEC(A) hydrolysis rate, s-1	Standart deviation	mEC(C) hydrolysis rate, s-1	Standart deviation	Ratio mutant/wt mEC(U)	Ratio mutant/wt mEC(A)
Ec WT	0.0010	0.00021	0.0035	0.00064	0.0005	0.00006	1	1
Ec Q771E	0.0014	0.00107	0.0042	0.00074			1.5	1.2
Ec F773V	0.0082	0.00165	0.0290	0.00232	0.0043	0.00030	8.6	8.1
Ec N792D	0.0063	0.00056	0.0125	0.00195			6.6	3.5
Ec I937T	0.0007	0.00031	0.0039	0.00071			0.8	1.1
Ec A940V	0.0105	0.0010	0.0260	0.00532			11.1	7.3
Ec A941F	0.0003	0.00004	0.0011	0.00013			0.3	0.3
Ec K1134G	0.0011	0.0002	0.0029	0.0004			1.2	0.8
Ec G1136Q	0.0056	0.00103	0.0111	0.00272	0.0023	0.00030	5.8	3.1
Ec V1141I	0.0001	0.00002	0.0056	0.00072			1.1	1.5
Ec A1142E	0.0007	0.0001	0.0082	0.00122			0.7	2.3
Ec D1143E	0.0012	0.0002	0.0051	0.00073			1.2	1.4
Ec F1145L	0.0028	0.00074	0.0033	0.00121			2.9	0.9
SspRNAP	0.0612	0.00814	0.1401	0.02423			63.1	39.3
SelRNAP	0.0502	0.00782	0.1212	0.00735			52.6	33.7
